# Targeted saliva metabolomics in Sjögren's syndrome

**DOI:** 10.1016/j.clinsp.2024.100459

**Published:** 2024-08-03

**Authors:** Giovanna Piacenza Florezi, Felippe Pereira Barone, Mario Augusto Izidoro, José Maria Soares-Jr, Claudia Malheiros Coutinho-Camillo, Silvia Vanessa Lourenço

**Affiliations:** a Stomatology Department, Faculdade de Odontologia, Universidade de São Paulo, São Paulo, SP, Brazil; b Tropical Medicine Institute, LIM-06, Faculdade de Medicina, Universidade de São Paulo, São Paulo, SP, Brazil; c Laboratório de Espectrometria de Massas do Hospital São Paulo, São Paulo, SP, Brazil; d Laboratório de Ginecologia Estrutural e Molecular (LIM-58), Disciplina de Ginecologia, Departamento de Obstetrícia e Ginecologia, Hospital das Clnicas HCFMUSP, Faculdade de Medicina da Universidade de São Paulo, São Paulo, SP, Brazil; e Centro Internacional de Pesquisa, A.C. Camargo Cancer Center, São Paulo, SP, Brazil

**Keywords:** Sjögren's Syndrome, Saliva, Metabolomics

## Abstract

**Objective:**

Sjögren's Syndrome (SS) is a chronic inflammatory autoimmune exocrinopathy, and although, the role of metabolism in the autoimmune responses has been discussed in diseases such as lupus erythematosus, rheumatoid arthritis, psoriasis and scleroderma. There is a lack of information regarding the metabolic implications of SS. Considering that the disease affects primarily salivary glands; the aim of this study is to evaluate the metabolic changes in the salivary glands' microenvironment using a targeted metabolomics approach.

**Methods:**

The saliva from 10 patients diagnosed with SS by the American-European consensus and 10 healthy volunteers was analyzed in an Ultra-high Performance Liquid Chromatograph Coupled Mass Spectrometry (UPLC-MS).

**Results:**

The results showed an increased concentration in SS of metabolites involved in oxidative stress such as lactate, alanine and malate, and amino acids involved in the growth and proliferation of T-cells, such as arginine, leucine valine and isoleucine.

**Conclusions:**

These results revealed that is possible to differentiate the metabolic profile of SS and healthy individuals using a small amount of saliva, which in its turn may reflect the cellular changes observed in the microenvironments of damaged salivary glands from these patients.

## Highlights

• The role of metabolism in Sjögren's Syndrome (SS) is still under discussion.
• Saliva can reflect the metabolic state of the microenvironments of salivary glands affected by SS.
• An increase in metabolites associated with oxidative stress can be observed.
• An increase in metabolites involved in the growth and proliferation of T-cells was also observed.

## Introduction

Primary Sjögren's Syndrome (pSS) is a chronic inflammatory autoimmune endocrinopathy. The disease is characterized by a lymphocytic infiltration upon epithelial activation and expression of autoantigens in exocrine salivary and lacrimal glands.^1^

The autoimmune response is triggered by epithelial cell activation, with the expression of Autoantibodies (anti-Ro), costimulatory proteins, immunoreactive MHC I/II and cellular adhesion molecules and the secretion of proinflammatory cytokines responsible for attracting lymphocytes, mainly CD4+T cells, including CD4+T Helper (Th). This cycle is perpetuated by the secretion of IFN-g, which induces the production of metalloproteinases and the activation of apoptotic mechanisms, leading to parenchyma destruction and stromal fibrosis.^2^ To be able to execute this series of events, the cells involved in this process must acquire the function of synthesizing biomolecules necessary to specific cellular functions, which occurs, for instance, as the bioenergetics demands of immune cells upon activation.^3^

Metabolism is the main responsible for acquiring, synthesizing and regulating substrates and products for complex biological reactions which fuel and allows the growth, proliferation, differentiation, and development of tissues and cells, culminating in the expression of their phenotypes.^4^ Based on the heterogeneity of immunological cells, their bioenergetics demands are usually related to tissue-specific functions. Cellular metabolism, accordingly, facilitates immune cell activity, as when inactivated, such cells are short in storage of nutrients. Following activation, immune cells increase growth and proliferation to engage in effector responses. These cells increase the uptake of amino acids, glucose and fatty acids from their environment.^4^, ^5^, ^6^, ^7^

It is well known that some autoimmune diseases, such as lupus erythematosus, rheumatoid arthritis, psoriasis and scleroderma present metabolic signatures, which lead to target personalized and more specific therapeutic options. These metabolic specificities are associated with target mechanisms of glucose metabolism (glycolysis, oxphos, pentose phosphate), lipid metabolism, aminoacid metabolism, rendering specific signatures to disease immunopathological phases, depending on the status of immune cells ‒ are quiescent, activated, or in memory state.^8^

Although metabolic pathways and their consequences have been long discussed in another autoimmune disease, there is a lack of information on metabolic implications in SS and their impacts on glandular and systemic alterations. Considering saliva an easy and non-invasive harvesting possibility, the authors evaluate a framework of the metabolic changes that occurred in immune cells present in the salivary glands’ microenvironment through the analysis of the salivary secretion of patients diagnosed with SS.

## Materials and methods

Paired samples with ten patients diagnosed according to the American-European consensus criteria for pSS and ten healthy volunteers including both genders with no age limitation were consecutively selected. Exclusion criteria comprised a confirmed history of other autoimmune diseases, head and neck radiotherapy, lymphoma, sarcoidosis, graft versus host disease, HIV and hepatitis B/C.

All patients underwent a minor salivary gland biopsy to evaluate the presence of lymphocytic infiltration and the focus. The labial salivary gland specimens were harvested according to the protocol established by Greenspan et al. (1974). The focus score was defined by the number of foci (> 50 lymphocytes) within 4 mm^2^ of salivary gland area.

The oral health of these patients was examined to exclude the interference of secondary inflammation derived from caries and/or periodontal diseases.

This study complies with the declaration of Helsinki and has received the approval of the Research Ethics Committee of the Dentistry and Medical Schools of the University of São Paulo (Protocols 2.097.226 and 2.119.193), and all participants were given and signed an informed consent.

### Salivary sample collection

Saliva collection was performed from 7 to 10 in the morning and the patients were told to refrain from eating, drinking, smoking and brushing their teeth for at least 2h before the procedure. Whole saliva was collected using a drooling method ‒ in which the patients were advised to lean their heads, without swallowing, and allow the accumulated saliva to drop in a sterile tube for a period of 5 minutes ‒ and kept in ice. Immediately following the collection, 1 % (v/v) of protease and phosphatase cocktail inhibitors (Sigma-Aldrich, St Louis, USA) were added, and the saliva was then centrifuged at 627 g, at a temperature of 4 °C for 20 minutes. The supernatant was divided into aliquots and stored at -20 °C until the analysis.

### Metabolite extraction, separation and detection

Proteins were precipitated by the addition of 300 mL of methanol and ethanol solution in a proportion of 1:1 (v/v) to 100 mL of sample and then centrifuged (2,500g) for 20 minutes at 4 °C, and the pellet was discarded. Then, 5 mL of each sample was added into a pool to yield an internal Quality Control (QC). Subsequently, 5 mL of phenylephrine and MES (2-(N-morpholino) ethanesulfonic acid (500 mM) was added to each sample as an internal standard sample control, and then the samples were put in chromatographic vials for analysis.

The selected targets comprehended metabolites involved in the energetic and oxidative stress metabolism, essential amino acids and hormones, described in Supplementary Table 1, with their identification numbers from the Human Metabolome Database (HMDB), PubChem and the Kyoto Encyclopedia of Genes and Genomes (KEGG).

The samples were analyzed in an ultra-high-performance liquid chromatograph Nexera System X2 coupled to a mass spectrometry (UPLC-MS) model 8060 (Shimadzu Co., Kyoto, Japan); sample injection rate of 2 mL, External Standards (ExStd) and Internal Standards (IStd) injection rate of 0.2 mL.

The chromatographic system counted with an ultra PFPP column (100 × 2.1 mm × 3 µm) used for the amino acids and biogenic amines analysis. Mobile phases A and B were composed of 100 % Mili-Q water and 100 % acetonitrile, respectively, with 0.1 % formic acid added to both. The pump flux adjusted to 0.3 mL/min and the column oven temperature stabilized at 40 °C.

Target metabolites separation and detection were realized using a linear gradient of B from 0.1 % to 1 % for 2 minutes, then a crescent B gradient to 99 % from 2 to 4 minutes, then held it for 1 minute (5 minutes total) and finally 1 minute to restore the column to its initial condition.

Ionization was conducted by an Electrospray Source (ESI), according to the parameters: ion spray source tension at 4.0 kV, nebulizing gas at 3.0 bar, drying gas at 10 mL/min and block heat temperature at 250 °C. The acquisition was realized in positive mode (ESI+) and the instrument sources Q1 and Q3 were configured to specifically analyze optimized ions by its own method, with a repeating time of 0.3 seconds. The data was collected by Multiple Reaction Measurement mode (MRM), by screening the precursor ion simultaneously with its ionic product.

Instrument control, data acquisition, and processing were carried out by LabSolutions software® (LCMS version 5.8), from Shimadzu Co., Japan, allowing control in real-time for each compound analyzed.

Identification of the compounds was realized from the comparison of the spectra obtained through the analyzed samples with the reference spectra acquired under the same experimental conditions, that is, the peaks of the analytes and standards will be integrated by the same software, using the same configurations. Thus, calibration curves were constructed to determine linearity, using external standards for each compound detected, generating quantitative values expressed in micro-Molar (mM). The data was then exported to an Excel format for further post-treatment and statistical analysis.

### Statistical analysis

The concentration data from the metabolites in mM was adjusted using the internal QC, according to a protocol established by Dunn et al., (2011),^9^ using the NOREVA tool, with low-order nonlinear Locally Estimated Smoothing function (LOESS),^10^ set in scale by Pareto method and normalized by logarithmic transformation.

The statistical analyses were conducted using MetaboAnalyst 5.0 (Chong et al., 2018), as well as IBM SPSS Statistics v. 28.0 (IBM Corp. Armonk, NY, USA), and GraphPad Prism version 9.0 (GraphPad Software, Inc).

A significance value of 5 % was corrected by a False Discovery Rate (FDR) of 5 % and a Fold Change (FC) of 2.0 was set. To determine differences between groups in continuous variables, the *t*-test was employed. Supervised classification using the Orthogonal Partial Least Squares Discriminant Analysis (OPLS-DA) method was employed for discriminant analysis among the analyzed groups. p-values were calculated to analyze the metabolite concentration differences between both groups by a *t*-test with two-tailed distribution using SPSS Statistics version 25.0 (IBM Corporation, Armonk, USA). The significance level was set at 95 %.

## Results

Among the selected patients, 8 were females with ages ranging around 41 years old. The sialometry test showed an average salivary flow of 0.26 mL/min. Xerostomia was reported by 9 patients and xerophthalmia by 8; objective tests showed an actual decrease in saliva secretion and lacrimal function in 7 and 6 patients, respectively. Serological tests revealed positive anti-SSA/Ro in 6 patients, anti-SSB/La in 9, rheumatoid factor in 6 individuals, and the Antinuclear Antibody (ANA) in all the patients (Table 1). The control group was composed of 9 females and 1 male, with an average age of 46.8 years.

**Table 1:** Epidemiological, clinical, and serological data, acquired from pSS patients’ charts.

Sample n°	Age	Gender	Xerophthalmia	Schirmer's test	Ocular Staining	Xerostomia	Scintigraphy	Focus Score	Anti-SSA/Ro	Anti-SSB/La	ANA	RF	Salivary Flow (mL/min)
1	54	F	+	-	-	+	+	2	+	+	1/640	+	0.04
2	31	F	+	-	-	+	-	5	-	+	1/160	-	0.206
3	56	F	+	+	+	+	+	3	-	+	1/320	+	0.45
4	30	F	-	+	+	+	+	2	-	+	1/320	-	0.38
5	54	F	+	+	-	+	+	2	+	+	1/80	+	0.31
6	20	F	-	-	-	-	-	3	+	+	1/320	+	0.38
7	52	F	+	-	+	+	+	4	+	-	1/160	+	0.26
8	39	F	+	+	+	+	+	3	+	+	1/80	-	0.1
9	31	M	+	+	+	+	-	3	-	+	1/160	+	0.18
10	43	M	+	+	+	+	+	3	+	+	1/160	-	0.16

ANA, Antinuclear Antibody; RF, Rheumatoid Factor; N/I, Not Informed.

*American College of Rheumatology/European League Against Rheumatism classification criteria for primary Sjogren's Syndrome.

From the targeted metabolites, the concentration of L-Asparagine, L-Cysteine, L-Tryptophan, R-Glutathione, Alpha-Ketoglutarate, Trimethylamine N-oxide (TMAO), Serotonin, DOPA, Adenosine, Adenine and Kynurenine could not be detected and quantified in the saliva from both groups.

**Fig. 1:**
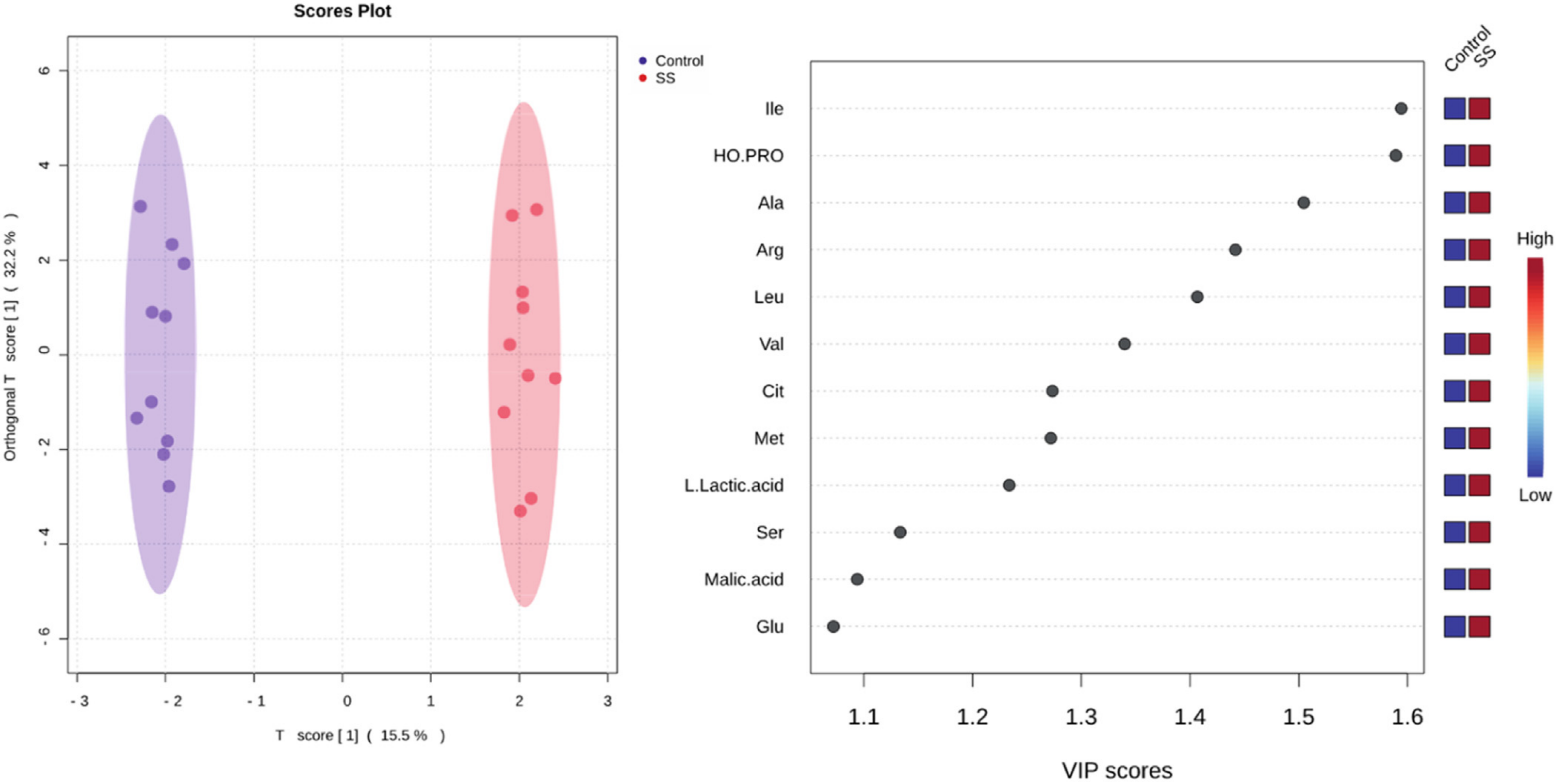
Graphic representation of the variation within and between groups, regarding the variables of the OPLS-DA analysis and the VIP scores of predictive components in the composition of the discriminant model between SS and control groups in the metabolomic analysis of saliva.

The statistical quality of the OPLS-DA model was considered substantial upon cross-validation (Q2 = 0.739, p < 0.05), with a clear separation observed between healthy volunteers and SS (Fig. 1). ROC curve showed 100 % of sensitivity and specificity (p = 0.0002).

Following the preliminary evaluation and visualization of the metabolomics data through multivariate analysis, the *t*-test allowed the observation of which metabolite had a statistically significant difference in concentration in the present samples, which can be observed in Table 2.

**Table 2:** Metabolite abundance from SSp and the healthy control group.

Metabolite	FC	Group	Mean	SD	CV	p-value (*t*-test)
Creatinine	126.50	CG	0.219	0.075	0.344	0.111
		SS	0.277	0.080	0.287	
Guanosine	476.37	CG	0.078	0.057	0.724	0.011^a^
		SS	0.372	0.325	0.875	
Thymine	136.17	CG	0.177	0.108	0.609	0.318
		SS	0.241	0.165	0.685	
L-Lactic acid	353.26	CG	0.097	0.079	0.819	0.024^a^
		SS	0.341	0.305	0.894	
Malic acid	18.73	CG	0.150	0.067	0.447	0.032^a^
		SS	0.282	0.166	0.588	
Succinic acid	192.79	CG	0.169	0.171	101.124	0.150
		SS	0.326	0.282	0.865	
Uric acid	0.91	CG	0.294	0.093	0.317	0.437
		SS	0.266	0.059	0.222	
Pyruvic acid	110.58	CG	0.264	0.040	0.152	0.684
		SS	0.292	0.210	0.719	
Phosphocreatine	0.84	CG	0.291	0.072	0.247	0.310
		SS	0.245	0.118	0.480	
Ornithine	0.97	CG	0.289	0.169	0.583	0.927
		SS	0.281	0.198	0.706	
L-Asparagine	0.93	CG	0.326	0.109	0.333	0.589
		SS	0.302	0.086	0.285	
L-lysine	152.22	CG	0.196	0.178	0.911	0.242
		SS	0.298	0.198	0.667	
L-Proline	166.19	CG	0.170	0.175	103.243	0.171
		SS	0.282	0.177	0.628	
L-Arginine	220.35	CG	0.138	0.068	0.492	0.010^a^
		SS	0.303	0.168	0.553	
L-Threonine	159.61	CG	0.226	0.066	0.290	0.045^a^
		SS	0.361	0.187	0.517	
L-Valine	205.63	CG	0.157	0.128	0.813	0.027^a^
		SS	0.322	0.176	0.545	
L-Glutamine	141.58	CG	0.208	0.174	0.839	0.310
		SS	0.294	0.195	0.663	
4-Hydroxyproline	188.79	CG	0.201	0.114	0.569	0.003^a^
		SS	0.379	0.120	0.315	
L-Leucine	267.28	CG	0.150	0.111	0.741	0.011^a^
		SS	0.401	0.258	0.643	
L-Phenylalanine	155.84	CG	0.188	0.152	0.809	0.186
		SS	0.293	0.188	0.640	
L-Methionine	266.09	CG	0.115	0.119	102.926	0.032^a^
		SS	0.306	0.231	0.755	
Citrulline	212.81	CG	0.146	0.158	108.157	0.066
		SS	0.312	0.215	0.691	
L-Histidine	150.84	CG	0.223	0.183	0.820	0.279
		SS	0.337	0.264	0.784	
L-Alanine	21.43	CG	0.154	0.139	0.905	0.019^a^
		SS	0.330	0.164	0.498	
L-Isoleucine	285.08	CG	0.154	0.118	0.766	0.005^a^
		SS	0.440	0.259	0.588	
L-Serine	193.27	CG	0.207	0.145	0.700	0.053
		SS	0.399	0.256	0.641	
Glycine	120.11	CG	0.228	0.177	0.773	0.566
		SS	0.274	0.175	0.637	
L-Tyrosine	114.87	CG	0.247	0.198	0.801	0.691
		SS	0.284	0.210	0.737	
L-Glutamic acid	189.48	CG	0.164	0.134	0.814	0.093
		SS	0.311	0.226	0.725	

FC, Fold Change; SD, Standard Deviation; CV, Coefficient of Variation.

a p < 0.05.

## Discussion

The knowledge of the metabolic products from the biological processes involved in the destruction of salivary glands in SS has a great appeal in the aid of understanding the disease physiopathology. Nevertheless, one of the most important features observed in these patients is the decrease of salivary flow caused by this salivary gland destruction; thus, the use of saliva in biochemical studies that require a larger amount of fluid is compromised. The present study, however, showed that using a minimal quantity of saliva is sufficient to observe relevant differences between healthy and impaired salivary flow.

The salivary test threshold must also consider the deterioration of saliva production with the progression of the pSS. Some studies, such as Fenoll-Palomares et al.,^11^ showed age- and sex-related variations in unstimulated whole saliva.^11^^,^^12^ Moreover, pSS usually occurs around menopause with a peak age between 40 and 45 years; the menopausal phase has physiologically reduced salivary flow and because of these factors, published studies have suggested an adjustment of the threshold for hyposalivation, modifying the cutoff to 0.2 mL/min to better fit the population menopausal.^11^, ^12^, ^13^

Multiple studies have been trying to use different techniques to establish a metabolic profiling of pSS. Herrala et al.,^14^ in metabolic salivary profiling comparing the inter and intra-individual variation using NMR spectroscopy, corroborates these findings revealed a higher concentration of alanine, although the observed levels suffered a high variation over time. Kageyama et al.,^15^ using gas chromatography, identified 88 metabolites in the saliva from pSS patients, however, while Herrala et al. showed an increased concentration of glycine in pSS, Kageyama et al. found the same metabolite in a significantly lower concentration in pSS patients, and in the present study, there was no significant difference between both groups.

Recently, Setti et al.^13^ detected approximately 50 metabolites in whole unstimulated saliva from a cohort of pSS patients analyzed by NMR. Their results indicated a higher abundance of amines, organic acids and 5-aminopentanoic acid and its lactam in the pSS group, showing that butyrate was higher in pSS patients than in HCs, while proline was higher in HCs; thus, the outcomes, found no difference from proline between pSS population vs. HC, disagree with results of Setti et al.

On the other hand, Li et al.^16^ demonstrated that the level of proline was elevated in saliva from pSS. This study identified a total of 38 metabolites, the most affected pathways included several amino acid metabolites, and the most important result showed that some metabolites regulated derived from tyrosine and phenylalanine metabolic pathways, suggesting a role in inflammatory injury in pSS disease.^16^

Therefore, these different outcomes could reflect the interference of sex, age, diet, lifestyle, drug effects, hormone levels, and stress resulting in a high variability of the metabolite concentration levels, even inside the pSS group. Furthermore, the wide application of MS technology is a consequence of its high sensitivity, which, permits qualitative exploration of the metabolome.^13^, ^14^, ^15^

Kageyama et al. suggested that the inflammation observed in salivary glands from pSS patients could interfere with the salivary metabolite profiles resulting in different disease phenotypes.^15^ The mechanisms of this interference, however, remain unclear, thus the authors discuss a variety of possibilities for these mechanisms based on the already described relationships from these metabolic reactions with pSS and autoimmune diseases.

The present results revealed a higher concentration of metabolites involved in the energetic metabolism such as lactate and alanine in pSS. Under normal circumstances, all cells rely on the production of ATP to guarantee the maintenance of their basic functions, which is acquired primarily by the glycolysis, Tricarboxylic Acid (TCA) cycle, and Oxidative Phosphorylation (OXPHOS) pathways; the last is dependent on oxygen availability. In the glycolysis process, glucose is metabolized into pyruvate, ATP, and NADH. Under aerobic conditions, pyruvate will enter the TCA cycle to be oxidized into CO_2_ and H_2_O, generating NADH, which will be oxidized by OXPHOS to generate ATP and NAD+.^12^

In anaerobic conditions, cells rely on the maintenance of glycolysis and the conversion of pyruvate into lactate, alanine, and NAD+, which will be secreted from the cells. However, when activated, Antigen Presenting Cells (APC) become dependent on glycolysis even in the presence of oxygen.^17^ This increases the concentration of lactate and Reactive Oxygen Species (ROS), as the activation of these cells also produces Nitric Oxide (NO), which ceases oxygen consumption by the mitochondria, completely inhibiting OXPHOS.^18^ These processes are part of the oxidative stress mechanisms, described in the pathophysiology of multiple rheumatological diseases, such as lupus erythematosus and rheumatoid arthritis; however, further investigation is needed concerning the role of oxidative stress in SS.^18^^,^^19^

The present results also showed an increased concentration of malate, suggesting that T and B-lymphocytes enhance both aerobic glycolysis and TCA cycle in a balanced manner. This balance may occur due to the suppression of Krebs cycle, by inhibiting malate conversion to oxaloacetate.^20^^,^^21^ Malate will be released from the mitochondria to the cytosol, then converted by the malic enzyme into pyruvate, which will either re-enter the Krebs cycle or participate in aerobic glycolysis.^22^, ^23^, ^24^

The preference for aerobic glycolysis occurs especially in T-Helper 1 (Th1), Th2 and Th17 lymphocytes, which generate enough ATP and biosynthetic intermediates due to a necessity of cell biomass availability to support a massive clonal expansion and differentiation from T-cells upon activation and to allow effector functions, like the secretion of IL-17.^25^^,^^26^

Amino acid availability plays an important role in T-lymphocytes activation and metabolism, corroborating the increased arginine availability in SS, since the lack of this amino acid leads to a suppression of the CD3ζ chain, impairing T-cell function by inhibiting proliferation.^27^, ^28^, ^29^

These study also revealed an increased concentration of leucine, valine and isoleucine in SS, sustaining the importance of a microenvironment availability of these amino acids in the growth and proliferation of T-cells by the activation of the Mechanistic Target of Rapamycin (mTOR).^30^, ^31^, ^32^, ^33^ Recently described in the salivary glands of pSS patients, mTOR is a serine/threonine protein kinase with two distinct complexes: mTOR Complex 1 (mTORC1) and 2 (mTORC2).^34^

It is established that mTORC1 promotes the shift from oxidative phosphorylation to glycolysis and increases the flux through the oxidative Pentose Phosphate Pathway (PPP). The last is essential to the production of R5P, essential in the synthesis of nucleic acids to support cell proliferation; and NADPH which, under normal circumstances, should promote a reducing environment and protection against oxidative stress by lipid biosynthesis.^30^^,^^35^

It was possible to distinguish the pSS group from the healthy control group, and these results confirmed that UPLC-MS is appropriate for screening pSS. A drawback of this study is the multiple treatment approaches for these patients, the different ages, and disease onset. For ethical reasons, the authors could not interrupt treatment and unfortunately, we could not enroll patients who were not receiving any kind of medication prior to the diagnosis due to its difficulties and frequent confusion with other autoimmune diseases. Further experiments are required to consolidate the diagnostic and prognostic potential of salivary metabolomics in primary Sjogren Syndrome.

## Conclusion

In conclusion, the present study showed that saliva might reflect the cellular changes observed in the microenvironments of damaged salivary glands from these patients. The results showed an increased concentration in pSS of metabolites involved in oxidative stress such as lactate, alanine and malate, and amino acids involved in the growth and proliferation of T-cells, such as arginine, leucine valine and isoleucine. This work was an incipient study for pSS metabolic profile analysis; further studies should expand the number of patient samples and validate the potential biomarkers.

The authors received financial support from FAPESP ‒ Fundação de Amparo à Pesquisa do Estado de São Paulo (2017/11806-7; 2018/00419-5; 2019/12702-6; 2021/13517-8) and declare no potential conflicts of interest with respect to the authorship and/or publication of this article.

## Declaration of competing interest

The authors declare no conflicts of interest.

## Appendix Supplementary materials

Image, application 1
