# Supplementary material for: Targeted saliva metabolomics in Sjögren's syndrome
Source: Clinics (Sao Paulo). 2024 Aug 3;79:100459. doi: 10.1016/j.clinsp.2024.100459 (PMC11334732; doi:10.1016/j.clinsp.2024.100459)
Supplement: Supplementary file 1 [file mmc1.docx]

**CLINICS-D-24-00222_Supplementary Material**

**Supplementary Table 1** Metabolite indexing descriptors for HMDB, PubChem and KEGG.

| **Metabolite** | **HMDB** | **PubChem** | **KEGG** |
| --- | --- | --- | --- |
| L-Aspartic acid | HMDB0000191 | 5960 | C00049 |
| L-Lysine | HMDB0000182 | 5962 | C00047 |
| L-Proline | HMDB0000162 | 145742 | C00148 |
| L-Arginine | HMDB0000517 | 6322 | C00062 |
| L-Threonine | HMDB0000167 | 6288 | C00188 |
| L-Valine | HMDB0000883 | 6287 | C00183 |
| L-Glutamine | HMDB0000641 | 5961 | C00064 |
| 4-Hydroxyproline | HMDB0000725 | 5810 | C01157 |
| L-Leucine | HMDB0000687 | 6106 | C00123 |
| L-Phenylalanine | HMDB0000159 | 6140 | C00079 |
| L-Methionine | HMDB0000696 | 6137 | C00073 |
| Citrulline | HMDB0000904 | 9750 | C00327 |
| L-Histidine | HMDB0000177 | 6274 | C00135 |
| L-Alanine | HMDB0000161 | 5950 | C00041 |
| L-Isoleucine | HMDB0000172 | 6306 | C00407 |
| L-Serine | HMDB0000187 | 5951 | C00065 |
| Glycine | HMDB0000123 | 750 | C00037 |
| L-Tyrosine | HMDB0000158 | 6057 | C00082 |
| L-Glutamic acid | HMDB0000148 | 33032 | C00025 |
| L-Ornithine | HMDB32455 | 389 | C01602 |
| Creatinine | HMDB0000562 | 588 | C00791 |
| Guanosine | HMDB0000133 | 6802 | C00387 |
| Thymine | HMDB0000262 | 1135 | C00178 |
| L-Lactic acid | HMDB0000190 | 107689 | C00186 |
| Malic acid | HMDB0000744 | 525 | C00711 |
| Succinic acid | HMDB0000254 | 1110 | C00042 |
| Uric acid | HMDB0000289 | 1175 | C00366 |
| Pyruvic acid | HMDB0000243 | 1060 | C00022 |
| Phosphocreatine | HMDB0001511 | 587 | C02305 |
| L-Asparagine | HMDB00168 | 3452 | C00152 |
| L-Cysteine | HMDB00574 | 3397 | C00097 |
| L-Tryptophan | HMDB00929 | 3378 | C00078 |
| R-Glutathione | HMDB0000125 | 3353 | C00051 |
| alpha-ketoglutarate | HMDB0000208 | 3328 | C00026 |
| Trimethylamine N-oxide (TMAO) | HMDB0000925 | 4338 | C01104 |
| Serotonin | HMDB0000259 | 4041 | C00780 |
| DOPA | HMDB0000181 | 3648 | C00355 |
| Adenosine | HMDB0000050 | 3512 | C00212 |
| Adenine | HMDB0000034 | 3447 | C00147 |
| Kynurenine | HMDB0000684 | 4855 | C01718 |
